# Polyostotic fibrous dysplasia with epiphyseal involvement of the proximal femur in a child: a case report and review of the literature

**DOI:** 10.3389/fped.2024.1505766

**Published:** 2025-01-20

**Authors:** Elio Paris, Giacomo De Marco, Oscar Vazquez, Sana Boudabbous, Christina Steiger, Romain Dayer, Dimitri Ceroni

**Affiliations:** ^1^ Faculty of Medicine, University of Geneva, Geneva, Switzerland; ^2^ Pediatric Orthopedic Unit, Pediatric Surgery Service, Geneva University Hospital, Geneva, Switzerland; ^3^ Radiology Department, Geneva University Hospital, Geneva, Switzerland

**Keywords:** fibrous dysplasia, proximal femur, pediatric, case report, epiphysis

## Abstract

Fibrous dysplasia (FD) is a benign medullary fibro-osseous anomaly that compromises the mechanical strength of bones, especially the long bones that bear strong mechanical stresses. It can lead to an inability to remodel immature bone into mature lamellar bone, resulting in inappropriate bone alignment in response to mechanical stresses. This case study describes a rare case of polyostotic FD presenting with an epiphyseal lesion of the proximal femoral head in its weight-bearing zone, accompanied by an unconventional femoral malrotation. The present case leads us to recommend that clinicians should not underestimate the occurrence of other deformities, such as the retrotorsion or flexion deformities that can compromise bone structure and the hip's biomechanics. Finally, the involvement of the epiphysis is probably more common than usually thought, introducing an additional complexity since juxta-articular lesions in weight-bearing joints may collapse, compromising articular congruence and function. To minimise this risk, bone scintigraphy and MRI should play a critical role in the patient's workup, evaluation, prognosis and follow-up.

## 1 Introduction

Fibrous dysplasia (FD) is a benign, non-hereditary, genetic bone disorder presenting as either an isolated skeletal lesion (its monostotic form) or affecting multiple bones (its polyostotic form) (1). The disease's incidence is estimated to be from 1 in 5,000 to 1 in 10,000 (2). FD is sometimes associated with single or multiple endocrinopathies, precocious puberty and cutaneous hyperpigmentation in McCune–Albright syndrome (3). Radiographically, it usually appears as a well-defined radiolucent medullary lesion that is irregular, mildly expansive and characterised by a hazy opacity typically described as “ground-glass” (4); it is usually designated as type IA according to the Lodwick classification (5). On long bones, FD can cause expansion of the bone edges, with cortical thinning and endosteal scalloping. The diaphysis is usually involved, but the metaphysis can also be affected (4). In very rare instances of the disease's polyostotic form, the epiphysis may be involved, especially in children (3, 6–10). These changes are usually visible on plain radiographs, but computed tomography and magnetic resonance imaging (MRI) are regularly performed to better investigate the tumour matrix and tumour expansion. This case study describes a rare case of polyostotic FD presenting with an epiphyseal lesion of the proximal femoral head in its weight-bearing zone, accompanied by an unconventional femoral malrotation.

## 2 Case report

A 10.5-year-old child underwent fixation surgery in a regional hospital for a pathological diaphyseal fracture of the left femur. A closed reduction of the fracture was performed and subsequently stabilised using two flexible retrograde intramedullary nails. After hardware removal, he was referred to our hospital centre at the age of 12 for a follow-up on his suspected fibrous dysplasia. On clinical examination, there was no leg length discrepancy, and no “café au lait” spots were noted on his skin. The patient tested positively during an anterior impingement (the FADIR test), with a severe restriction in internal rotation at his left hip related to his femoral retroversion.

**Figure 1:**
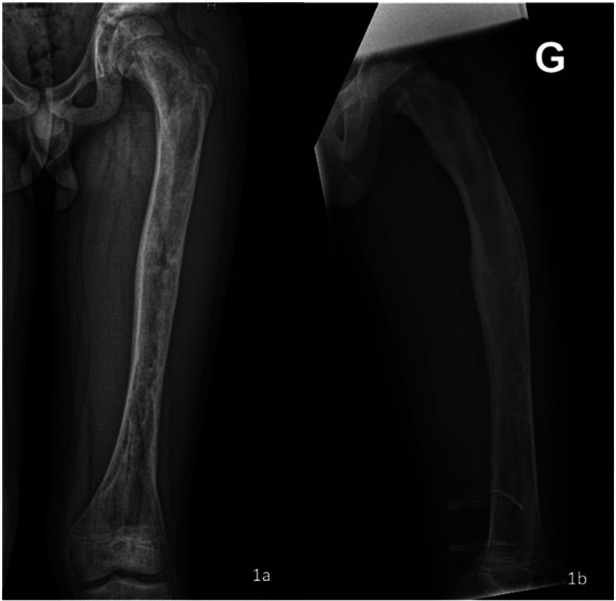
X-ray images of the left femur after removal of osteosynthetic materials: the frontal coronal plane image revealed a variety of radiolucent lesions in the diaphysis, with sclerotic edges, scalloping and a “ground-glass” appearance **(a)** sagittal plain radiographs showed an increased sagittal radius of curvature and retrotorsion of the proximal femur **(b).**

X-ray images of the left femur revealed various radiolucent lesions in the diaphysis, with sclerotic edges, scalloping and a ground-glass appearance (Figure 1a). The patient's plain radiographs showed no coxa vara or shepherd's crook deformities but revealed an increased sagittal radius of curvature and retrotorsion of the proximal femur (Figure 1b). The patient's growth plates were still open at the level of the proximal and distal femur.

**Figure 2:**
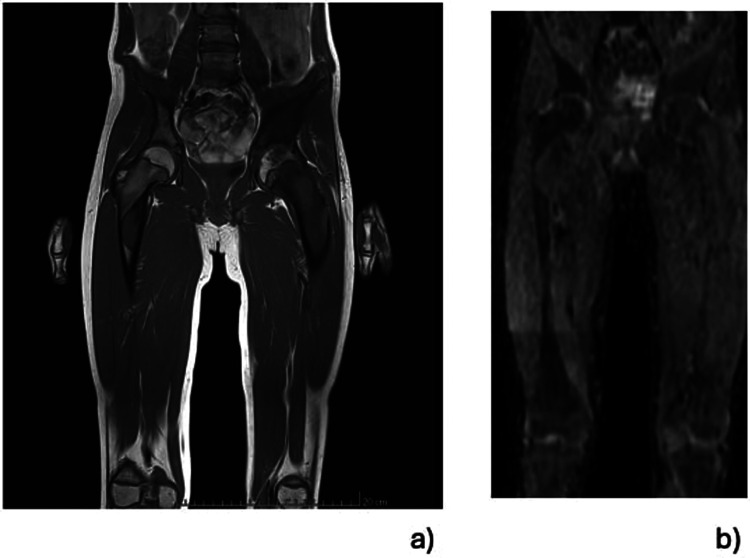
Selected total-body MRI at the level of the pelvis and both femurs using low signal intensity on T1-weighted images and demonstrating the foci of FD affecting the epiphysis, the femoral neck, the diaphyseal regions of the left femur and the same hip's acetabulum. A lesion is visible in the subtrochanteric region of the right femur **(a)**. There was a high signal intensity without diffusion restriction on the fat-suppressed T2-weighted images enhanced with gadolinium **(b).**

**Figure 3:**
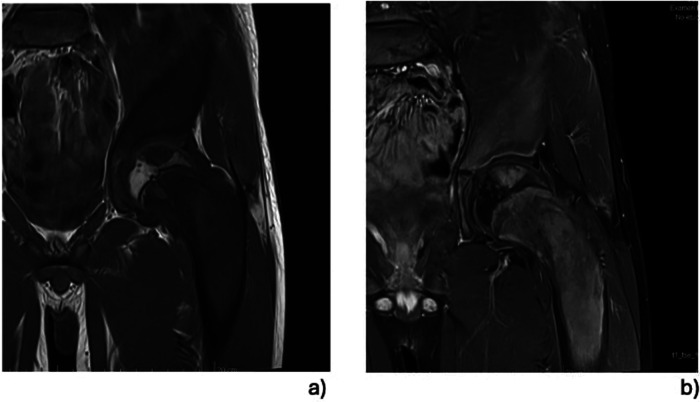
MRI focusing on left proximal femur lesions: the focus of the FD of the proximal epiphysis and the significant involvement of the femoral neck can be seen precisely. The examination did not demonstrate transphyseal diffusion of the FD, and the lesions seemed not to originate from the epiphyseal growth plate. All the lesions were characterised by a low signal intensity on T1-weighted images **(a)**, but these were significantly better in the T1-weighted images enhanced with gadolinium **(b).**

**Figure 4:**
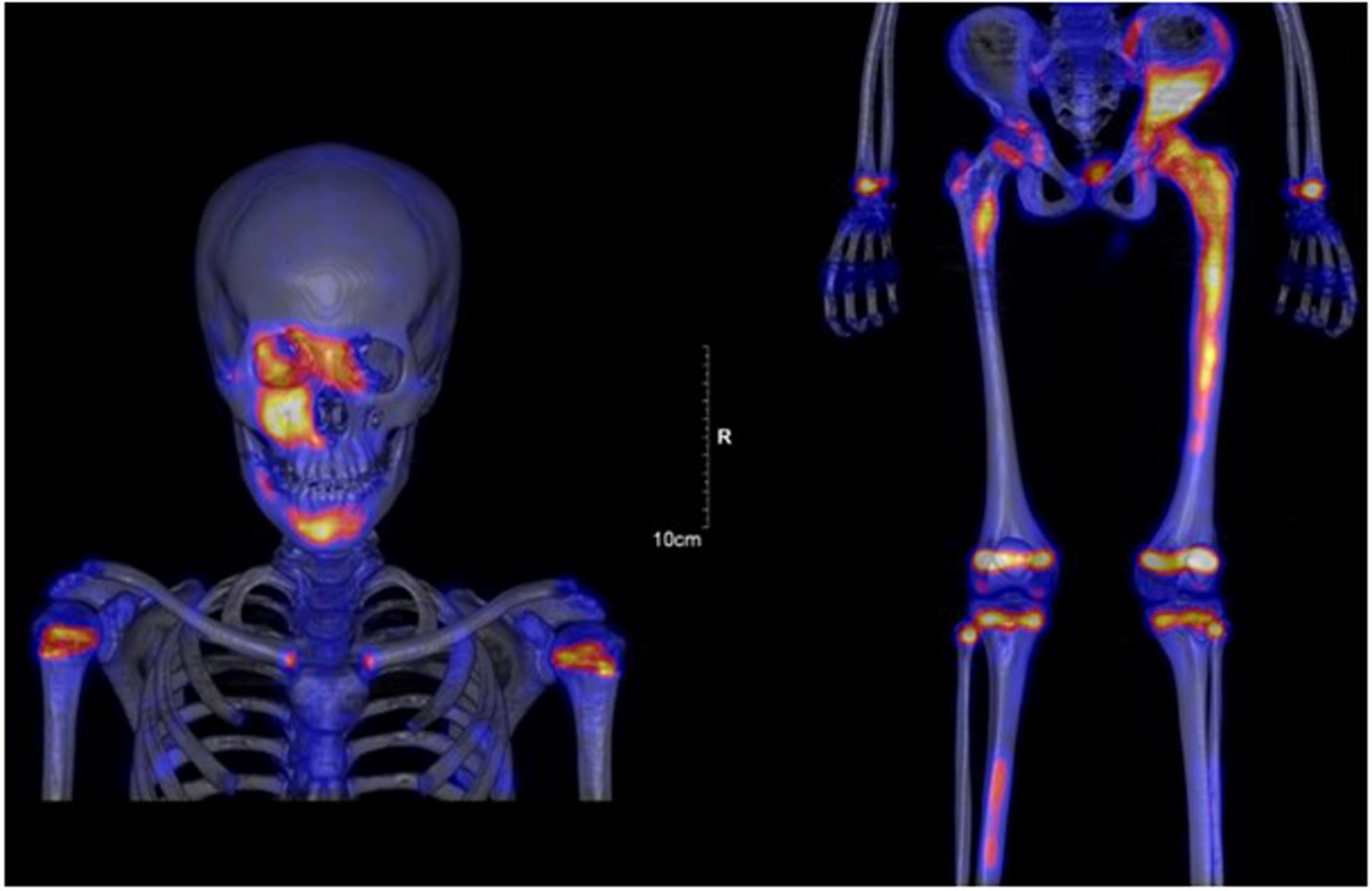
Scintigraphy confirmed polyostotic hyperfixation and demonstrated an unknown parasymphyseal involvement of the mandible with extension into its right branch.

Whole body MRI confirmed the diagnosis of polyostotic FD, displaying FD foci in the left femur, right tibia, left fibula, left calcaneus, both iliac wings, maxillary bone, and the sphenoid wing (Figure 2). The lesions on the left femur were in the proximal epiphysis, the proximal metaphysis and along the diaphysis. MRI demonstrated a low signal intensity in T1-weighted images (Figure 3a) and a high signal intensity in fat-suppressed T2-weighted images, also showing strong gadolinium enhancement (Figure 3b) without diffusion restriction (Figure 2b). There was no significant bone marrow or soft tissue enhancement. At the femoral head, the lesion was juxta-articular, with no subchondral bone collapse. Scintigraphy, added to assess skull involvement, confirmed the polyostotic hyperfixation and demonstrated an unknown parasymphyseal involvement of the mandible with an extension into its right branch (Figure 4). Finally, blood tests enabled us to exclude endocrine dysfunctions.

*Timeline:*

• Occurrence of a pathological fracture of the left femur in August 2023
• Closed reduction of the femoral fracture and stabilization by elastic intramedullary nailing in August 2023.
• Removal of osteosynthesis material in March 2024.
• Patient referred to our institution for further treatment in April 2024.
• Additional radiographic assessment between April and June 2024 (scintigraphy, bone scan, MRI, and echography of the genital tract).
• Blood analysis: April 2024.

## 3 Discussion

FD is a pathological condition that leads to an inability to remodel immature bone into mature lamellar bone, resulting in inappropriate bone alignment in response to mechanical stresses (6). Histologically, fibroblast proliferation will result in excessive fibrous tissue replacing normal calcium hydroxyapatite in the osteoid matrix (11). As previously noted, FD is classified into two types: the monostotic form affects a single bone, while polyostotic FD is characterised by the involvement of multiple bones. Polyostotic FD is frequently accompanied by manifestations of syndromes such as McCune–Albright syndrome (3) or Mazabraub syndrome (12), where it is associated with endocrine abnormalities and overproduction of melatonin in the skin (3) or with intramuscular myxomas (12), respectively. Most endocrinopathies present during FD revolve around hyperthyroidism, hyperparathyroidism, acromegaly, diabetes mellitus, and Cushing syndrome.

FD is the result of a mutation in the guanine-nucleotide alpha stimulating-GNAS gene. It seems to be recognised that the chronological timing of the mutation's appearance is responsible for a somatic mosaicism that determines the extent of the disease and its clinical manifestations (13). Mutations that occur at early stages of embryogenesis typically result in the widespread distribution of bone lesions (1). Conversely, mutations occurring at later stages of embryogenesis result in a more localised distribution (1). The involvement of a gene mutation is why FD cannot occur spontaneously and why the monostotic form of FD never progresses to the polyostotic form (1).

As a rule, when either monostotic or polyostotic FD occurs in long bones, such as the tibia, femur or humerus, it typically affects the diaphysis or metaphysis. Several authors have postulated that FD bone lesions usually spare the epiphysis (3, 14–16), and, when present, epiphyseal involvement depends, above all, on the patient's age. In fact, the literature suggests that diaphyseal and metaphyseal lesions can expand with growth and could even result in the involvement of the epiphysis after physeal closure in adults (7, 14–17). On the contrary, however, cases of FD with epiphyseal involvement before puberty are quite rare since only seven cases were found in previous reports involving paediatric populations (3, 6–10). Nixon and Condon postulated that FD in children might originate from a fibro-osseous aberration occurring in the epiphyseal growth plate, with a subsequent bidirectional extension into the epiphysis and the metaphysis (10). According to them, the lesion's extension across the epiphyseal growth plate supports this hypothesis (10).

Involvement of the proximal femoral epiphysis, as in our patient, has been described only in 2 cases in the paediatric population, even in polyostotic forms of FD (8). The present case is rich in information and leads to several realisations. Firstly, it confirms that the risk of a pathological fracture in FD patients varies according to the patient's age, with a major predisposition to fractures of the femur. Fractures are most prevalent between the ages of 6 and 10, and peak incidence is estimated to be 0.4 fractures per FD patient per year (1). Secondly, our case demonstrated that multiplanar proximal femoral deformity can occur in patients with FD. Typically, it is now recognised that femurs affected with FD in their proximal third will develop coxa vara deformities, leading to the characteristic “shepherd's crook” deformity (18–22). In our patient, the deformity occurred in the sagittal plane (a proximal femoral flexion deformity) and in the horizontal plane (a proximal femoral retrotorsion). Moreover, it is interesting to note that the above-cited deformities are recognised as generating femoroacetabular impingement. Thus, even though almost one-third of femurs affected by FD develop a typical coxa vara deformity, it also appears important to look out for deformities in all three planes and, thus, for clinical signs of femoroacetabular impingement. Thirdly, our case did not validate the pathophysiological hypothesis which suggests that FD may originate from a fibro-osseous aberration in the epiphyseal growth plate, with a subsequent extension into both the epiphysis and metaphysis. Instead, we have the impression that the distribution of lesions is random, with a predominance of diaphyseal and metaphyseal locations. This highlights the importance of doing a full radiological work-up using MRI to rule-out the presence and characteristics of multiple foci of bone lesions. Due to their insignificant appearance, one could imagine that epiphyseal lesions might be overlooked and underdiagnosed. Hence, patients with polyostotic FD should be systematically investigated for epiphyseal lesions (10), since epiphyseal lesions due to FD are structurally weak and introduce additional complexity since they can induce serious deformities into the articular surface. Indeed, juxta-articular lesions in weight-bearing joints can collapse leading to a loss of proper joint congruence.

Further radiological monitoring also remains essential because cystic changes are occasionally seen in FD lesions, with secondary transformations into aneurysmal bone cysts (9, 10, 23, 24). Even worse, FD lesions can degenerate into high-grade sarcoma, with an incidence of 0.5% in monostotic FD and 4% in McCune–Albright syndrome (24, 25). The most common forms of malignant degeneration, in decreasing order of frequency, are osteosarcoma, fibrosarcoma and chondrosarcoma. MRI is also crucial for assessing bone and soft tissue invasion and for guiding a percutaneous biopsy for a final diagnosis.

It is very common for general physician to be the first specialist consulted for FD, whatever its form, even though some children with McCune–Albright because of their non-orthopedic symptoms, such as skin pigmentation or precocious puberty. Patients' monostotic lesions are very frequently diagnosed incidentally on radiographs taken for unrelated symptoms and they must be referred to an orthopedic specialist, even if they are asymptomatic. Thus, radiological investigations, particularly bone scintigraphy and MRI, play a critical role in the identification, prognostic evaluation, and follow-up of osseous complications in paediatric patients with polyostotic FD.

## 4 Conclusion

Polyostotic fibrous dysplasia (FD) is a benign medullary fibro-osseous anomaly that compromises the mechanical strength of bones, especially the long bones that bear strong mechanical stresses. As a result, these may inappropriately align, particularly at the proximal femur. Deformities typically occur in the femur's frontal plane, ranging from the coxa vara deformity to the well-known “shepherd's crook” deformity. The present case leads us to recommend that clinicians should not underestimate the occurrence of other deformities, such as the retrotorsion or flexion deformities that can compromise bone structure and the hip's biomechanics. Finally, the involvement of the epiphysis is probably more common than usually thought, introducing an additional complexity since juxta-articular lesions in weight-bearing joints may collapse, compromising articular congruence and function. In order to minimise this risk, bone scintigraphy and MRI should play a critical role in the patient's workup, evaluation, prognosis and follow-up.

## Data Availability

The original contributions presented in the study are included in the article/Supplementary Material, further inquiries can be directed to the corresponding author.
